# Nomograms predicting Overall Survival and Cancer-specific Survival for Synchronous Colorectal Liver-limited Metastasis

**DOI:** 10.7150/jca.46155

**Published:** 2020-08-27

**Authors:** Yuqiang Li, Wenxue Liu, Lilan Zhao, Cenap Güngör, Yang Xu, Xiangping Song, Dan Wang, Zhongyi Zhou, Yuan Zhou, Chenglong Li, Qian Pei, Fengbo Tan, Haiping Pei

**Affiliations:** 1 Department of Gastrointestinal Surgery, Xiangya Hospital, Central South University, Changsha, China.; 2 Department of General Visceral and Thoracic Surgery, University Medical Center Hamburg-Eppendorf, Hamburg, Germany.; 3 Department of Cardiology, Xiangya Hospital, Central South University, Changsha, China.; 4 Department of Rheumatology, Guangdong Provincial People's Hospital, Guangdong Academy of Medical Sciences, Guangzhou, China.; 5 Department of Thoracic surgery, Fujian Provincial Hospital, Fuzhou, China.

**Keywords:** Nomogram, colorectal cancer, liver metastasis, overall survival, cancer-specific survival

## Abstract

**Background:** Colorectal cancer (CRC) ranks as the third most frequent cancer type and the second leading cause of cancer-related death worldwide. The liver is the most common metastatic site of CRC with 20%-34% of patients suffering synchronous liver metastasis. Patients with colorectal liver-limited metastasis account for one-third of deaths from colorectal cancer. Moreover, some evidence indicated that CRC patients with synchronous liver disease encounter a worse prognosis and more disseminated disease state comparing with metastatic liver disease that develops metachronously.

**Methods:** Data in this retrospective analysis were extracted from the Surveillance, Epidemiology, and End Results (SEER) database. Nomograms were constructed with basis from a multivariate Cox regression analysis. The prognostic nomograms were validated by C-index, time-dependent receiver operating characteristic (ROC) curve, decision curve analysis (DCA) and calibration curves.

**Results:** A total of 9,958 CRC patients with synchronous liver-limited metastasis were extracted from the SEER database during 2010-2016. Both overall survival (OS) and cancer-specific survival (CSS) were significantly correlated with age, marital status, race, tumor location, pathological grade, histologic type, T stage, N stage, surgery for primary tumor, surgery for liver metastasis, chemotherapy and CEA. All of the significant variables were used to create the nomograms predicting OS and CSS. C-index values, time-dependent ROC curves, DCA curves and calibration curves, proved the superiority of the nomograms.

**Conclusions:** Our research investigated a national cohort of almost 10,000 patients to create and verify nomograms based on pathological, therapeutic and demographic features to predict OS and CSS for synchronous colorectal liver-limited metastasis (SCLLM). The nomograms may act as an excellent tool to integrate clinical characteristics to guide the therapeutic choice for SCLLM patients.

## Introduction

Colorectal cancer (CRC) ranks as the third most frequent cancer type and the second leading cause of cancer-related death worldwide 1. The liver is the most common metastatic site of CRC with 20%-34% of patients suffering synchronous liver metastasis 2, 3. Meanwhile, hepatic metastasis is now the leading cause of death in CRC patients 4. Patients with colorectal liver-limited metastasis account for one-third of deaths from colorectal cancer 5. Moreover, some evidence indicated that CRC patients with synchronous liver disease encountered a worse prognosis and more disseminated disease state comparing with metastatic liver disease that develops metachronously 6. Accordingly, this study focused on synchronous colorectal liver-limited metastasis (SCLLM).

Notwithstanding that technologies and therapeutic strategies have progressed over the last several decades, the survival of CRC patients with synchronous liver-limited metastasis still remains unsatisfactory. It is urgent to identify prognostic factors for patients with SCLLM. A nomogram, a simple graphical representation combining and quantifying all independent prognostic factors 7, plays an increasingly important role in medical research and clinical practice. Large public databases, like the Surveillance, Epidemiology, and End Results (SEER) database provide available, authentic and reliable data to explore clinical issues.

The purpose of this study was to construct nomograms predicting overall survival (OS) and cancer-specific survival (CSS) for patients with SCLLM based on the SEER database.

## Materials and Methods

### Patients

Data in this retrospective analysis were extracted from the SEER database. The SEER Program of the National Cancer Institute is an authoritative source of information on cancer incidence and survival in the United States (U.S.) that is updated annually. The definition of SCLLM is colorectal cancer with liver-limited metastases at the time of diagnosis. Therefore, colorectal adenocarcinoma patients (ICD-O-3: 8140, 8144, 8145, 8201, 8210, 8211, 8213, 8253, 8255, 8260, 8261, 8262, 8263, 8310, 8323, 8480, 8481, 8490) with liver metastasis were collected from the period 2010-2016, resulting in 32,353 patients in total. Exclusion criteria: diagnosed at autopsy or death certificate (n=26); survival months is 0 (n=3289); lack of positive histology (n=489); status of lung, bone and brain is yes, unknown or N/A (n=8488); T0, T4NOS, Tx, N1NOS, N2NOS, M1b, M1 and blank(s) in AJCC stage (n=10103). The final study sample contained 9,958 patients.

For each patient, the following data was acquired: age at diagnosis, marital status, insurance, gender, race, grade, histological type, T stage, N stage, regional nodes examined (RNE), CEA, surgery for primary tumor, surgery for hepatic metastasis, perineural invasion (PNI), radiotherapy and chemotherapy. We defined colectomy with RNE ≥12 as standard colectomy and colectomy with RNE <12/NOS as simplified colectomy. All patients were randomly separated into two groups (training group, n = 6639 and validation group, n = 3319).

### Follow-up and outcome

The follow-up cutoff was December 31, 2016. The endpoint of this study was OS and CSS. OS was computed from the time of diagnosis to the time of death due to any cause or the time of last follow-up with the patient still alive. CSS was computed from the time of diagnosis to the time of death attributed to colorectal cancer or still alive at last follow-up censored. The OS and CSS curves were evaluated using the Kaplan-Meier method and compared using the log-rank test.

### Statistical Analysis

An odds ratio (OR) and a 95% confidence interval (CI) were evaluated by univariable and multivariate Cox regression model. Variables with significant differences in univariate analysis were included in the Cox regression model for multivariate analysis. Nomograms were constructed with basis from the multivariate analysis results, using R 3.6.1 software (Institute for Statistics and Mathematics, Vienna, Austria; http://www.r-project.org/). The prognostic nomograms were validated by a C-index, time-dependent receiver operating characteristic (ROC) curve, decision curve analysis (DCA) and calibration curves. Statistical analyses were performed with IBM SPSS statistics trial ver. 22.0 (IBM, Armonk, NY, USA). All reported *p*-values lower than 0.05 were considered significant.

## Results

### Patient Characteristics

A total of 9,958 CRC patients with synchronous liver-limited metastasis were extracted from the SEER database for the period from 2010-2016. Characteristics of the target population were summarized in **Table 1.** A total 6,639 patients were divided into a training cohort and 3,319 into a validation cohort. Insurance covered 94.45% of SCLLM patients. The majority of patients were elderly (≥60 years), married, and white. The right colon (41.33%) was the most common tumor location in SCLLM. Interestingly, patients with T3 accounted for 57.41%, which was more than the ratio of T4 (28.67%). In addition, positive lymph nodes (68.77%) and CEA (58.24%) were detected in most patients. The median OS and CSS were 17-month and 18-month respectively.

Most SCLLM patients underwent the surgery for primary tumor, including 68.95% of cases that received the colectomy with an RNE of more than 12 and 14.19% of patients accepted simplified colectomy. Meanwhile, hepatic surgery was performed for only 19.49% of SCLLM patients. Lastly, 2,532 (25.43%) patients missed chemotherapy in this study.

### Independent prognostic factors for OS and CSS

Independent predictors were identified by univariable and multivariable Cox regression analyses. The multivariate Cox regression model was further applied to analyze the qualified variables in univariable one. As shown in **Table 2 and 3**, both of OS and CSS were significantly correlated with age, marital status, race, tumor location, pathological grade, histologic type, T stage, N stage, surgery for primary tumor, surgery for liver metastasis, chemotherapy and CEA.

**Figure 1:**
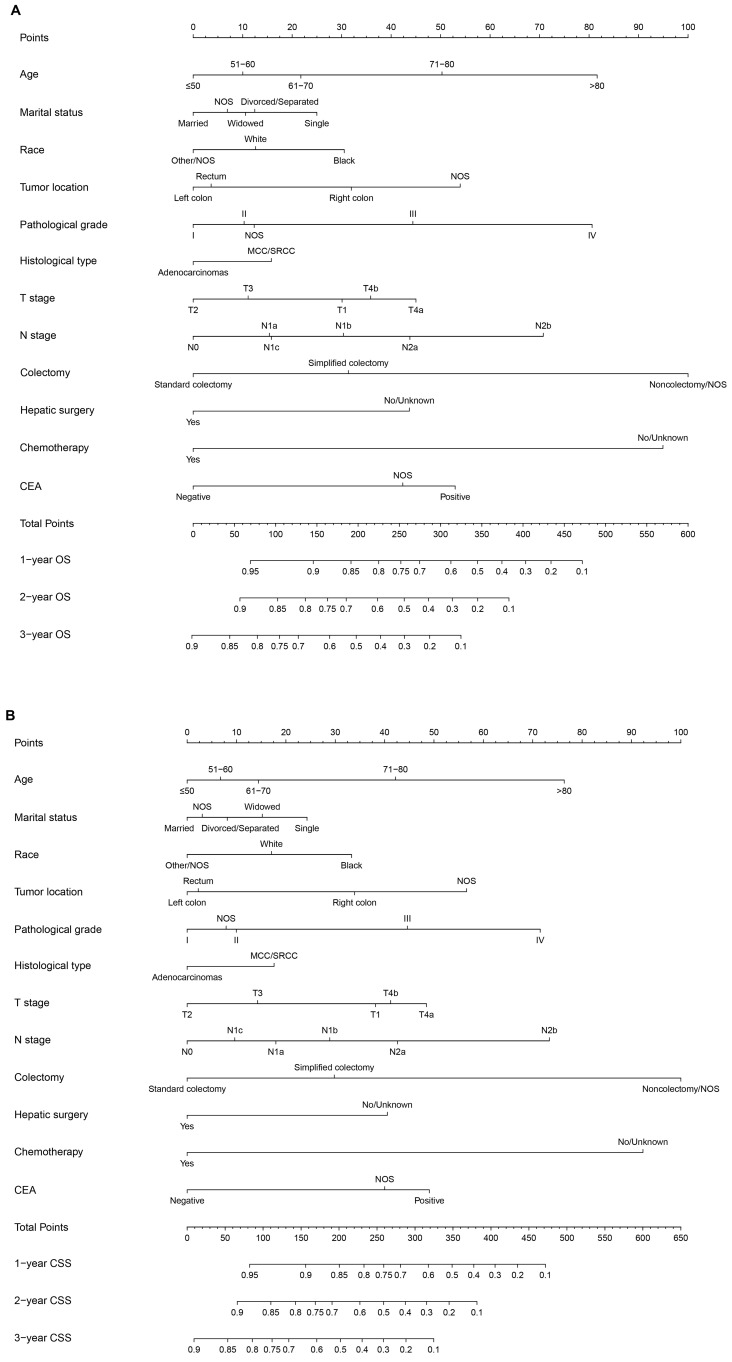
** A.** Nomogram of predicting OS for patients with SCLLM; **B.** Nomogram of predicting CSS for patients with SCLLM.

All of the significant variables were used to create the nomograms for OS and CSS. The prognostic nomogram for 1-, 2-, and 3-year OS was shown in **Figure 1A.** The prognostic nomogram for 1-, 2-, and 3-year CSS was shown in **Figure 1B.** By adding up the scores related to each variable and projecting total scores to the bottom scales, we were easily able to calculate the estimated 1-, 2-, and 3-year OS and CSS probabilities.

### Calibration and Validation of Prognostic Nomograms

**Figure 2:**
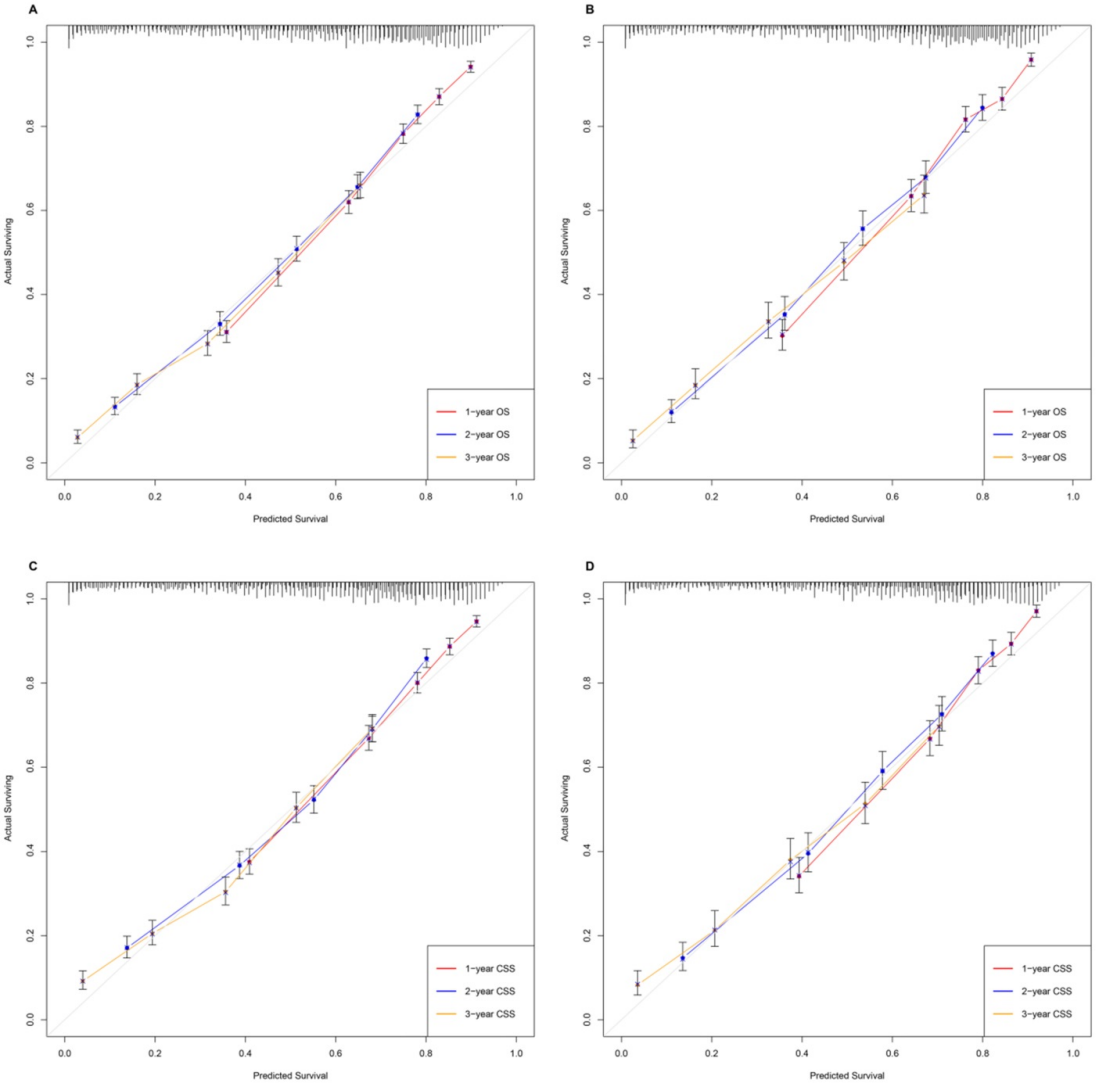
The calibration curves, without obviously deviations from the reference line, illustrated optimal agreement between model prediction and actual observations for 1-, 2-, 3-year OS and CSS. **A.** Predicting patients' OS at 1-year, 2-year, 3-year in the training group. **B.** Predicting patients' OS at 1-year, 2-year, 3-year in the validation group. **C.** Predicting patients' CSS at 1-year, 2-year, 3-year in the training group. **D.** Predicting patients' CSS at 1-year, 2-year, 3-year in the validation group.

Various methods, including C-index values, time-dependent ROC curves, decision curve analysis (DCA) and calibration curves, were utilized to evaluate the discriminating superiority of nomograms. The C-indexes proved that the nomograms provided favorable predictive accuracy. The nomogram predicting OS obtained 0.744 (95%CI: 0.736-0.752) and 0.749 (95%CI: 0.738-0.760) regarding the C-index in the training and validation group, respectively. While the C-index values of the nomogram predicting CSS were 0.741 (95%CI: 0.732-0.750) and 0.753 (95%CI: 0.741-0.766) in the training and validation group, respectively (**Table 4**). Besides, the calibration curves were able to visually illustrate the relationship between actual probability and predicted probability. As shown in **Figure 2**, the calibration curves, without obvious deviations from the reference line, illustrated the optimal agreement between model prediction and actual observations for 1-, 2-, 3-year OS and CSS.

**Figure 3:**
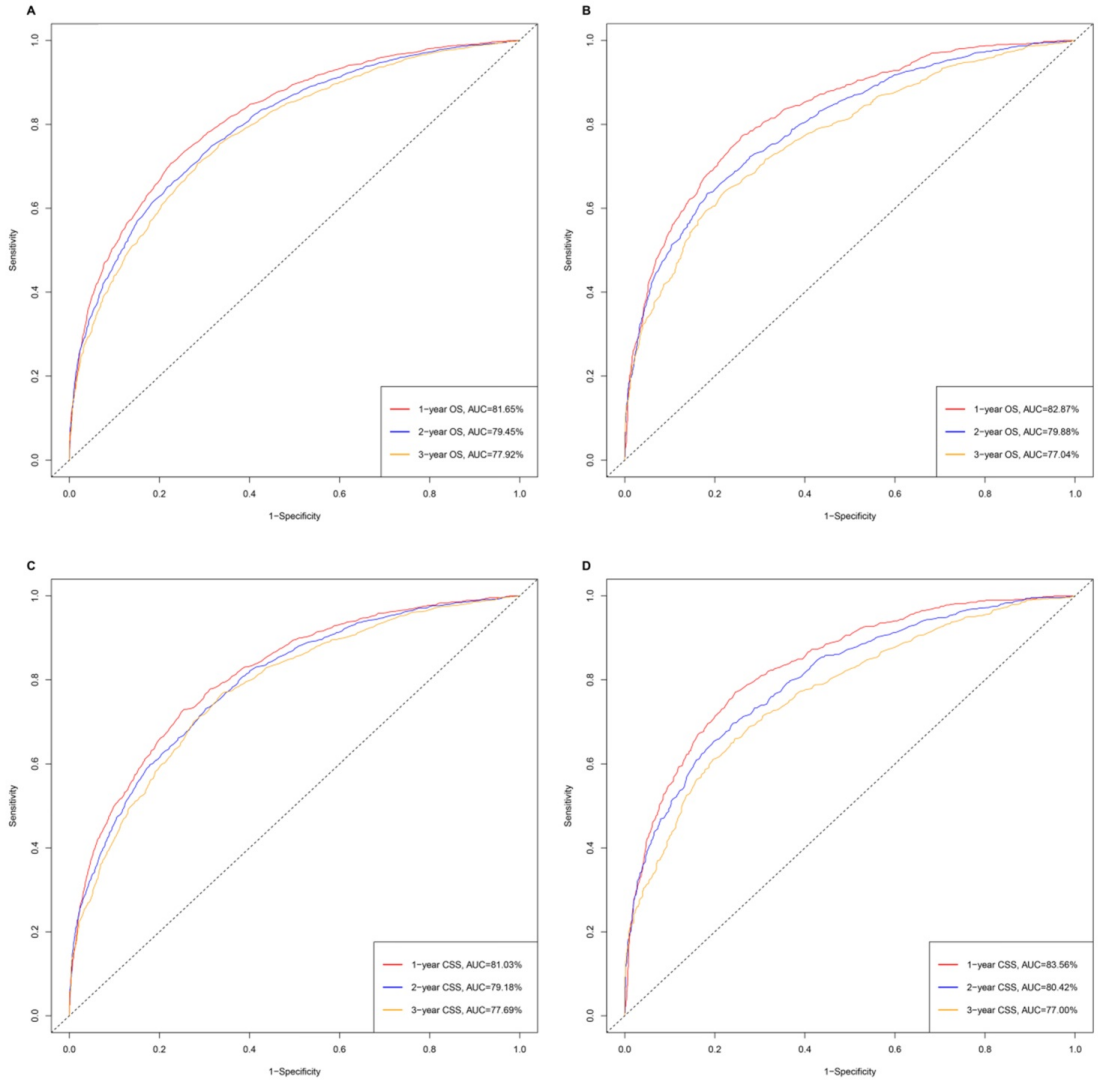
The time-dependent ROC curves of nomograms. **A.** The AUC values of ROC were 81.65%, 79.45% and 77.92% regarding nomograms predicting 1-, 2- and 3- year OS in training cohort**. B.** The 1-, 2-, and 3-year AUC values of the nomogram for OS were 82.87%, 79.88% and 77.04% in validation cohort**. C.** The AUC values of ROC were 81.03%, 79.18% and 77.69% regarding nomograms predicting 1-, 2- and 3- year CSS in training cohort**. D.** The 1-, 2-, and 3-year AUC values of the nomogram for CSS were 83.56%, 80.42% and 77.00% in validation cohort.

The time-dependent receiver operating characteristic (ROC) has been used widely to display sensitivity and specificity in predictive models. The area under the curve (AUC) values of ROC were 81.65%, 79.45% and 77.92% regarding for nomograms predicting 1-, 2- and 3- year OS, respectively, in the training cohort. While the 1-, 2-, and 3-year AUC values of the nomogram for OS were 82.87%, 79.88% and 77.04%, respectively, in the validation cohort. Similarly, the nomogram of CSS obtained the outstanding AUC values in training (AUC=81.03% for 1-year CSS; AUC=79.18% for 2-year CSS and AUC=77.69% for 3-year CSS) and the validation group (AUC=83.56% for 1-year CSS; AUC=80.42% for 2-year CSS and AUC=77.00% for 3-year CSS) (**Figure 3**).

**Figure 4:**
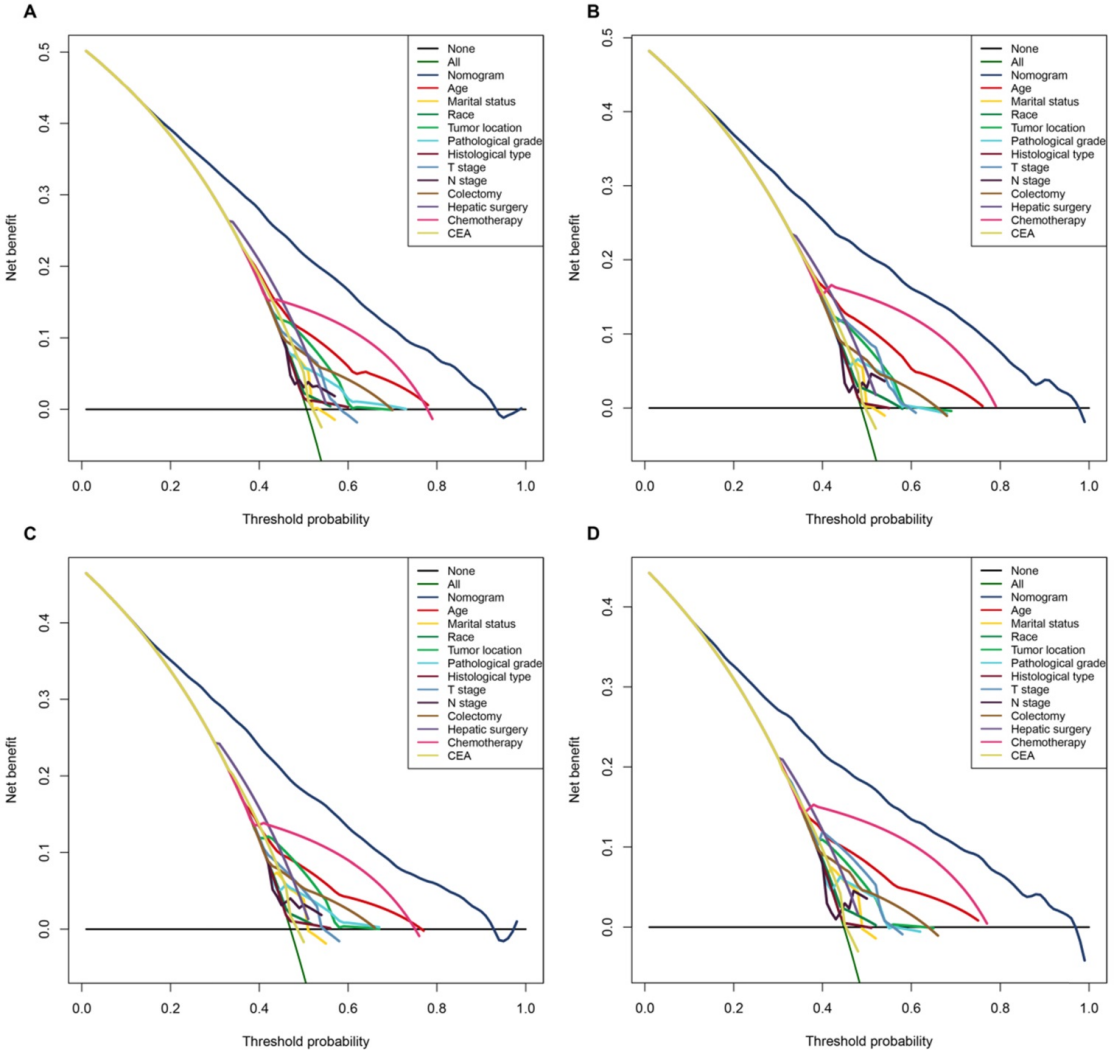
The decision curve analysis (DCA) demonstrated that the nomograms owned excellent net benefits and was superior to the any single prognostic factors across the wider range of reasonable threshold probabilities in OS and CSS. **A.** The DCA of the nomogram and all prognostic factors for OS in the training cohort. **B.** The DCA of the nomogram and all prognostic factors for OS in the validation cohort. **C.** DCA of the nomogram and all prognostic factors for CSS in the training cohort. **D.** The DCA of the nomogram and all prognostic factors for CSS in the validation cohort.

Moreover, in terms of clinical utility, DCA demonstrated that the nomograms, provided excellent net benefits and were superior to the any single prognostic factors across the wider range of reasonable threshold probabilities in OS and CSS (**Figure 4**).

### Performance of the Nomograms in Stratifying on the Basis of Risk Scores

**Figure 5:**
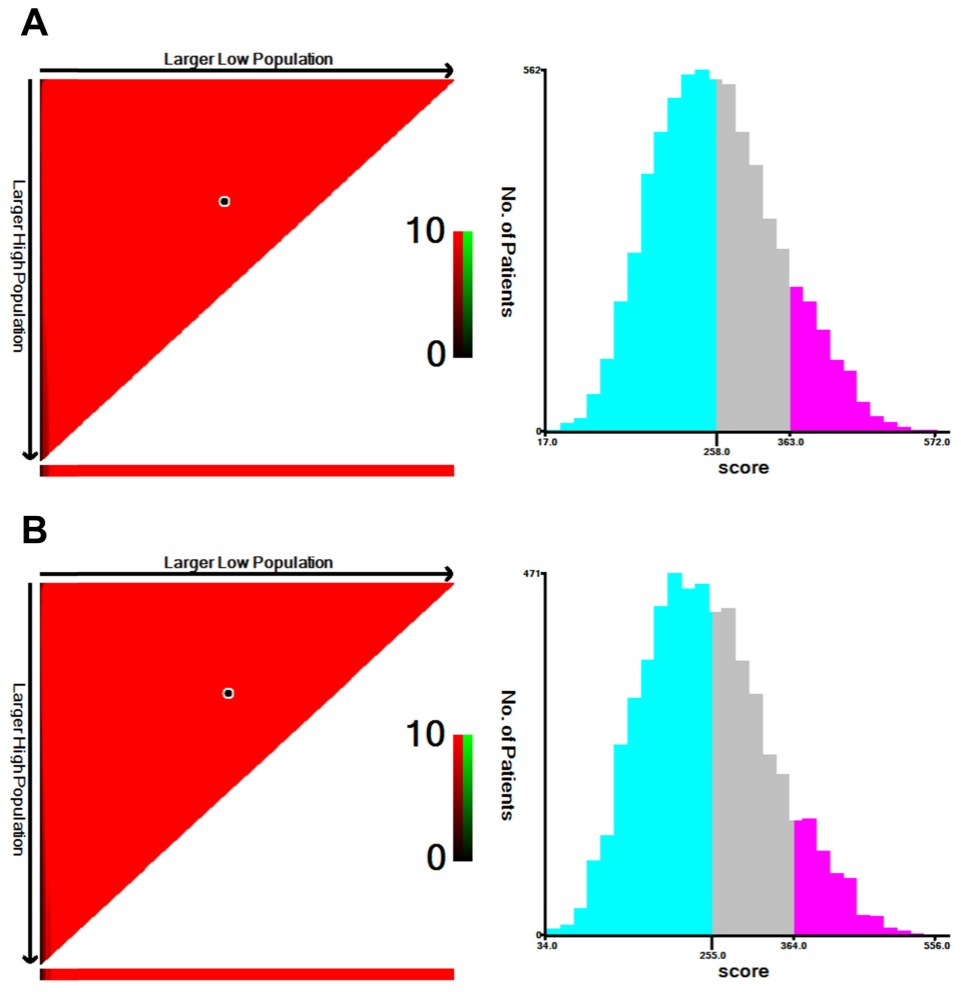
The cut-off values were calculated by using X-tile based on the total scores of patients in the training cohort. **A.** According to the cut-off values of the nomogram for OS, SCLLM were divided into low-risk (score < 258), moderate-risk (258 ≤ score < 363) and high-risk (score ≥ 363). **B.** According to the cut-off values of the nomogram for CSS, SCLLM were divided into low-risk (score < 255), moderate-risk (255 ≤ score < 364) and high-risk (score ≥ 364).

The prognostic scores of all independent predictors were assigned on the basis of the established nomogram, and optimal cut-off values were calculated by using X-tile based on the total scores of patients in the training cohort 8. According to the cut-off values of the nomogram for OS, SCLLM were divided into low-risk (score < 258), moderate-risk (258 ≤ score < 363) and high-risk (score ≥ 363) (**Figure 5**). Similarly, patients were classified into three subgroups based on total score (< 255, 255 to 364, and ≥ 364) for CSS (**Figure 5**).

**Figure 6:**
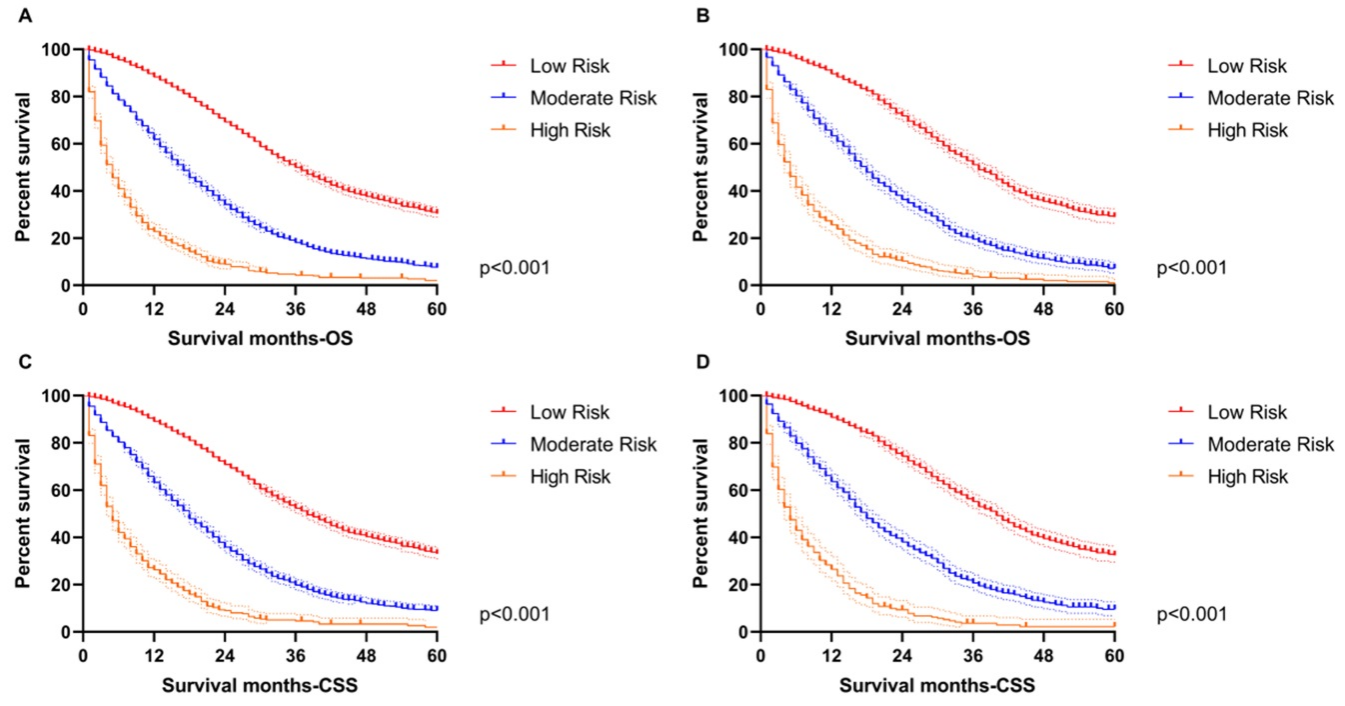
The survival analysis in the subgroup. **A.** The low-risk cohort owned the longest median OS (36-month) followed by the moderate-risk cohort (17-month OS) and high-risk cohort (5-month for OS) in the training group. **B.** The low-risk cohort owned the longest median OS (37-month) followed by the moderate-risk cohort (18-month OS) and high-risk cohort (5-month for OS) in the validation group. **C.** The low-risk cohort owned the longest median CSS (38-month) followed by the moderate-risk cohort (18-month CSS) and high-risk cohort (5-month for CSS) in the training group. **D.** The low-risk cohort owned the longest median CSS (40-month) followed by the moderate-risk cohort (18-month OS) and high-risk cohort (5-month for OS) in the validation group.

Additionally, the Kaplan-Meier survival curves were subsequently delineated and are shown in **Figure 6.** In the training group, the low-risk cohort owned the longest median OS (36-month) and CSS (38-month), followed by the moderate-risk cohort (17-month OS and 18-month CSS) and the high-risk cohort (5-month for OS and CSS). We obtained consistent results in the validation cohort (low-risk group: 37-month median OS and 40-month median CSS; moderate-risk group: 18-month median OS and CSS; high-risk group: 5-month median OS and CSS).

**Figure 7:**
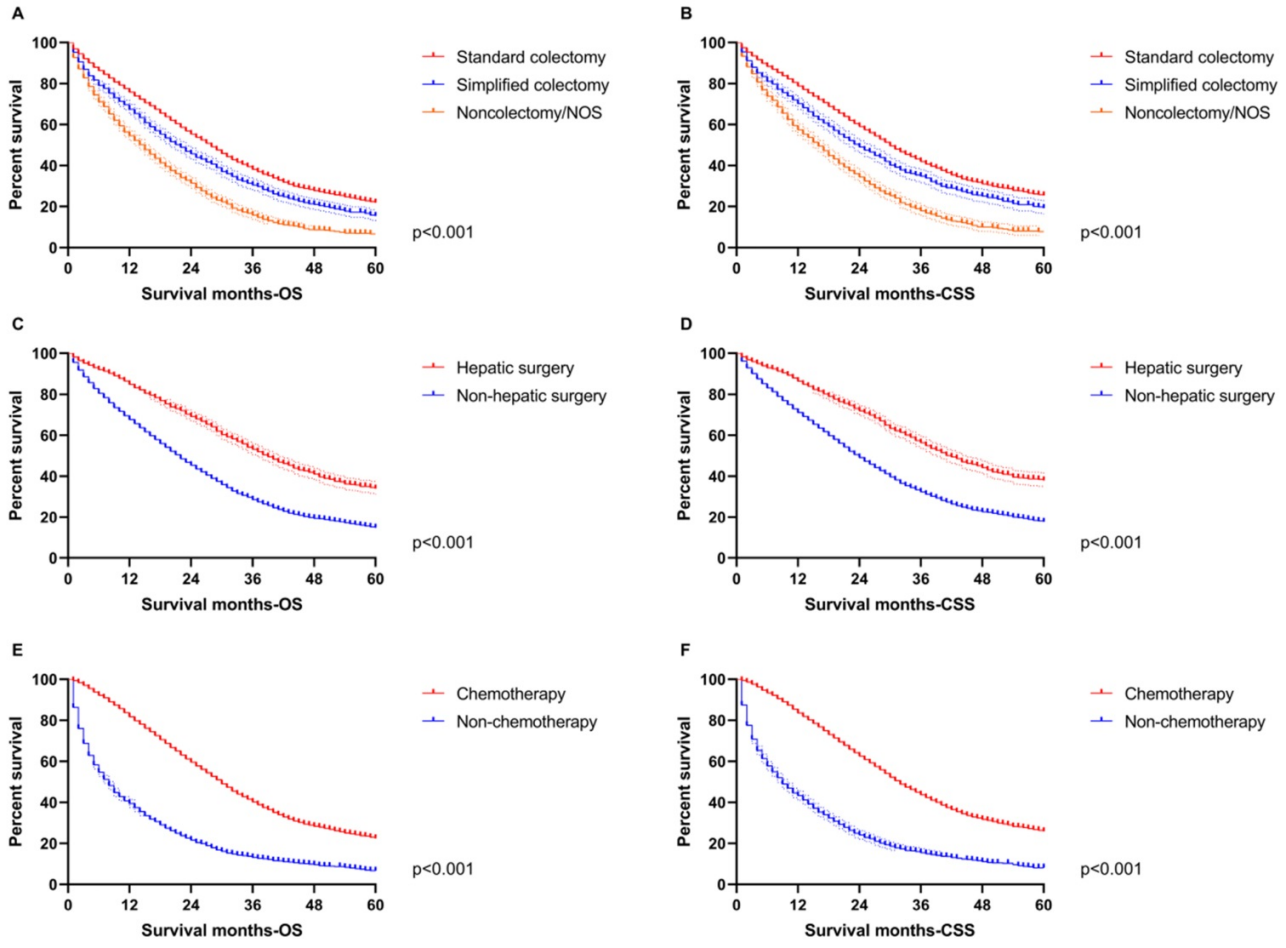
The survival analysis for therapeutic features in the total population. **A.** The difference of OS among standard colectomy (median OS: 28-month), simplified colectomy (median OS: 22-month) and non-colectomy/NOS (median OS: 15-month). **B.** The difference of CSS among standard colectomy (median CSS: 30-month), simplified colectomy (median CSS: 24-month) and non-colectomy/NOS (median CSS: 16-month). **C.** The difference of OS between hepatic surgery (median OS: 39-month) and non-hepatic surgery (median OS: 22-month). **D.** The difference of CSS between hepatic surgery (median CSS: 42-month) and non-hepatic surgery (median CSS: 24-month). **E.** The difference of OS between chemotherapy (median OS: 30-month) and non-chemotherapy (median OS: 8-month). **F.** The difference of CSS between chemotherapy (median CSS: 32-month) and non-chemotherapy (median CSS: 9-month).

In order to highlight the role of therapeutic variables, survival curves were also drawn to indicate the benefit from treatment based on the total population in this study. All primary surgery, hepatic operation and chemotherapy improved OS and CSS distinctly (*p*<0.001, **Figure 7**), which was consistent with the nomograms.

## Discussion

This study provided a significant contribution through the use of a large cohort of patients with SCLLM who were treated in the U.S. from 2010 to 2016 to construct nomograms predicting OS and CSS, which were capable of providing individualized estimates of potential survival benefit and can aid individualized management decisions for SCLLM. Other scoring systems, including various clinicopathological factors, have been developed to evaluate survival for SCLLM 9, however, the limitations of such risk scoring systems included a lack of reproducibility when applied at other institutions 10. The SEER database, with cancer incidence and survival data from population-based cancer registries covering approximately 34.6% of the population from U.S. 11, provides available, authentic and reliable data, which can make up for limitations regarding perfect reproducibility. Meanwhile, the comprehensive nomograms with an absolute net benefit advantage over any single prognostic factor in DCA curves provided excellent value for clinical practice. Moreover, the superior accuracy, sensitivity and specificity of nomograms predicting OS and CSS were able to ensure effectiveness in clinical practice.

Chemotherapy is recommended for all CRC patients with synchronous metastatic diseases. The nomograms demonstrated the ginormous risk in SCLLM patients without chemotherapy, which was similar in the survival curves. However, an optimal chemotherapy regimen remains controversial, along with the order of surgery and chemotherapy. Regrettably, this study failed to explore further due to limitations of the SEER database. Moreover, several researches suggested that surgical resection should not be performed unless all known tumors can be completely removed (R0 resection), because incomplete resection or debulking (R1/R2 resection) did not provide survival beneficial for CRC patients with metastatic diseases 12, 13. Did patients with SCLLM really not get any survival beneficial from the separate primary resection? The multivariable Cox regression analyses believed that surgical resection for the primary tumor could be used as an independent predictor. Moreover, the proportion of primary resections was significantly higher than that of hepatic surgery in our study. We then delineated the survival curves to definitely compare the difference among non-colectomy, standard and simplified colectomy in patients without hepatic surgery (**Figure S1**). All the evidences indicated that SCLLM patients could receive survival benefit from the separate resection for a primary tumor. Results from one study also suggested that there may be some benefit in both OS and PFS from resection of the primary in the setting of unresectable colorectal metastases 14. Separate analyses of the National Cancer Data Base also identified a survival benefit of primary tumor resection in this setting 15. More importantly, colectomy with RNE ≥12 provided a longer OS and CSS than one without, reminding surgeons that lymph node dissection cannot be ignored in colorectal cancer with synchronous liver-limited metastasis.

Age was also an important prognostic factor in this study. Increasing age was accompanied by an elevated risk score, especially in patients over 70-year-old. Marital status was also able to affect the OS and CSS of patients with SCLLM. Single persons suffered the greatest risk, but persons with a stable marriage status owned the lowest risk. It may be that the company of a significant other is supportive. In addition, the different survival among ethnic groups should also be given attention.

A growing body of data indicated that primary tumor location can be a prognostic factor in metastasis colorectal cancer 16-18, which was consistent with the nomograms in this study. Increasing research reported multitudinous differences between right and left colon cancer, involving embryonic origin, molecular genetics, pathological type as well as demographic characteristics such as gender and age 19-23. Moreover, cetuximab and panitumumab, as monoclonal antibodies directed against EGFR, confer little benefit to patients with metastatic colorectal cancer if the primary tumor originated on the right side 16-18. Therefore, some scholars suggested that primary tumor sidedness is a surrogate for the non-random distribution of molecular subtypes across the colorectum and, enables a better biologic understanding of the observed difference in response to EGFR inhibitors 6.

The roles of pathological grade, histological type and CEA in the nomograms were in line with our notions. However, T and N stages were not completely consistent with our knowledge. The nomograms reminded that SCLLM patients with early T stage should be given more attention because the risk score of T1 was even more than that of T2-3. Additionally, patients with negative regional lymph nodes, but positive tumor deposits (TD) in specific site were divided into a N1c stage 6, that obtained an equal or even a lower risk score comparing with N1a. Therefore, it is worth considering whether the risk degree of TD needs to be redefined in the TNM stage system for patients with synchronous metastases. Moreover, PNI was included as a high-risk factor for systemic recurrence 6, but did not affect the survival of patients with metastasis.

Currently, there are different definitions of synchronous metastasis for colorectal cancer 24-26. Although some definitions include metastases detected up to 6 months following diagnosis 25, 26, most include detection at or before diagnosis or surgery of the primary tumor 24. Moreover, Adam R, et al. also believed that synchronous metastasis for colorectal cancer should be defined as synchronously detected 27. There are still some shortcomings in this study: (1) further validation is necessary due to the typical limits of a retrospective study; (2) some important information is missing in the SEER database, such as Ras and B-raf; and (3) a lack of detailed data precluded an ability to compare the pros and cons of chemotherapy regimens. However, the excellent clinical value should not be masked by these shortcomings.

## Conclusion

Our research investigated a national cohort of almost 10000 patients to create and verify nomograms based on pathological, therapeutic and demographic features to predict OS and CSS for SCLLM. The nomograms may act as an excellent tool to integrate clinical characteristics to guide the therapeutic choice for SCLLM patients.

## Supplementary Material

Supplementary figure S1.

## Figures and Tables

**Table 1:** Characteristics of patients with SCLLM in the training and validation group

Characteristics	Total (n=9958)		Training group (n=6639)		Validation group (n=3319)	
	N	%	N	%	N	%
**Gender**						
Female	4239	42.57%	2828	42.60%	1411	42.51%
Male	5719	57.43%	3811	57.40%	1908	57.49%
**Age (years)**						
≤50	1734	17.41%	1178	17.74%	556	16.75%
51-60	2398	24.08%	1580	23.80%	818	24.65%
61-70	2692	27.03%	1816	27.35%	876	26.39%
71-80	1899	19.07%	1243	18.72%	656	19.76%
>80	1235	12.40%	822	12.38%	413	12.44%
**Marital status**						
Married	5351	53.74%	3598	54.19%	1753	52.82%
Single	1794	18.02%	1188	17.89%	606	18.26%
Divorced/Separated	1129	11.34%	729	10.98%	400	12.05%
Widowed	1198	12.03%	796	11.99%	402	12.11%
NOS	486	4.88%	328	4.94%	158	4.76%
**Insurance**						
Yes	9405	94.45%	6273	94.49%	3132	94.37%
No/unknown	553	5.55%	366	5.51%	187	5.63%
**Race**						
White	7556	75.88%	5040	75.92%	2516	75.81%
Black	1531	15.37%	1010	15.21%	521	15.70%
Other/NOS	871	8.75%	589	8.87%	282	8.50%
**Tumor location**						
Right colon	4116	41.33%	2777	41.83%	1339	40.34%
Left colon	3367	33.81%	2199	33.12%	1168	35.19%
Rectum^ †^	2294	23.04%	1549	23.33%	745	22.45%
NOS	181	1.82%	114	1.72%	67	2.02%
**Pathological grade**						
I	377	3.79%	259	3.90%	118	3.56%
II	6637	66.65%	4426	66.67%	2211	66.62%
III	1750	17.57%	1181	17.79%	569	17.14%
IV	378	3.80%	240	3.62%	138	4.16%
Unknown	816	8.19%	533	8.03%	283	8.53%
**Histological type**						
Adenocarcinomas	9397	94.37%	6259	94.28%	3138	94.55%
MCC/SRCC	561	5.63%	380	5.72%	181	5.45%
**T stage**						
T1	996	10.00%	670	10.09%	326	9.82%
T2	390	3.92%	266	4.01%	124	3.74%
T3	5717	57.41%	3786	57.03%	1931	58.18%
T4a	1800	18.08%	1218	18.35%	582	17.54%
T4b	1055	10.59%	699	10.53%	356	10.73%
**N stage**						
N0	3110	31.23%	2081	31.35%	1029	31.00%
N1a	1264	12.69%	855	12.88%	409	12.32%
N1b	1805	18.13%	1215	18.30%	590	17.78%
N1c	224	2.25%	151	2.27%	73	2.20%
N2a	1660	16.67%	1104	16.63%	556	16.75%
N2b	1895	19.03%	1233	18.57%	662	19.95%
**Colectomy**						
Standard colectomy	6866	68.95%	4567	68.79%	2299	69.27%
Simplified colectomy	1413	14.19%	946	14.25%	467	14.07%
Non-colectomy/NOS	1679	16.86%	1126	16.96%	553	16.66%
**Hepatic surgery**						
Yes	1941	19.49%	1285	19.36%	656	19.76%
No/unknown	8017	80.51%	5354	80.64%	2663	80.24%
**Radiotherapy**						
Yes	963	9.67%	638	9.61%	325	9.79%
No/Unknown	8995	90.33%	6001	90.39%	2994	90.21%
**Chemotherapy**						
Yes	7426	74.57%	4958	74.68%	2468	74.36%
No/Unknown	2532	25.43%	1681	25.32%	851	25.64%
**CEA**						
Negative	1351	13.57%	886	13.35%	465	14.01%
Positive	5800	58.24%	3899	58.73%	1901	57.28%
NOS	2807	28.19%	1854	27.93%	953	28.71%
**PNI**						
Negative	5971	59.96%	3977	59.90%	1994	60.08%
Positive	2188	21.97%	1493	22.49%	695	20.94%
NOS	1799	18.07%	1169	17.61%	630	18.98%
OS (months)	17 (7-31)		17 (7-31)		18 (8-32)	
CSS (months)	18 (8-32)		18 (8-31)		18 (8-32)	

MCC: mucinous cell carcinoma; SRCC: signet ring cell carcinoma; RNE: regional nodes examined; PNI: perineural invasion; NOS: not otherwise specified. †: Rectum includes Rectosigmoid junction.

**Table 2:** Univariable and multivariable Cox regression model analyses of OS for nomogram

Characteristics	Univariable analysis				Multivariable analysis			
	OR	95% CI lower	95% CI upper	*p*-value	OR	95% CI lower	95% CI upper	*p*-value
**Gender**				0.118				
Female		Reference				NA		
Male	0.952	0.895	1.013	0.118				
**Age (years)**				<0.001				<0.001
≤50		Reference				Reference		
51-60	1.137	1.027	1.260	0.014	1.095	0.987	1.214	0.086
61-70	1.311	1.188	1.447	<0.001	1.225	1.108	1.355	<0.001
71-80	1.846	1.666	2.045	<0.001	1.597	1.434	1.778	<0.001
>80	3.294	2.951	3.677	<0.001	2.124	1.874	2.408	<0.001
**Marital status**				<0.001				<0.001
Married		Reference				Reference		
Single	1.238	1.139	1.345	<0.001	1.259	1.155	1.372	<0.001
Divorced/Separated	1.150	1.041	1.271	0.006	1.123	1.015	1.243	0.024
Widowed	1.887	1.722	2.067	<0.001	1.102	0.998	1.218	0.056
NOS	1.141	0.989	1.316	0.071	1.065	0.923	1.230	0.389
**Insurance**				0.405				
Yes		Reference				NA		
No/unknown	0.944	0.825	1.081	0.405				
**Race**				<0.001				<0.001
White		Reference				Reference		
Black	1.215	1.119	1.320	<0.001	1.179	1.082	1.285	<0.001
Other/NOS	0.890	0.795	0.996	0.042	0.892	0.797	1.000	0.049
**Tumor location**				<0.001				<0.001
Right colon		Reference				Reference		
Left colon	0.645	0.600	0.692	<0.001	0.743	0.689	0.800	<0.001
Rectum^ †^	0.682	0.630	0.738	<0.001	0.787	0.719	0.862	<0.001
NOS	1.372	1.104	1.705	0.004	1.227	0.985	1.527	0.068
**Pathological grade**				<0.001				<0.001
I		Reference				Reference		
II	0.942	0.801	1.108	0.471	1.097	0.931	1.292	0.269
III	1.418	1.195	1.683	<0.001	1.498	1.257	1.785	<0.001
IV	1.903	1.539	2.353	<0.001	2.066	1.661	2.568	<0.001
Unknown	1.325	1.097	1.599	0.003	1.122	0.925	1.360	0.243
**Histological type**				<0.001				0.018
Adenocarcinomas		Reference				Reference		
MCC/SRCC	1.329	1.175	1.504	<0.001	1.165	1.027	1.322	0.018
**T stage**				<0.001				<0.001
T1		Reference				Reference		
T2	0.436	0.358	0.531	<0.001	0.771	0.623	0.953	0.016
T3	0.559	0.507	0.616	<0.001	0.850	0.746	0.969	0.015
T4a	0.822	0.735	0.918	0.001	1.158	1.002	1.339	0.048
T4b	0.808	0.712	0.916	0.001	1.066	0.922	1.232	0.387
**N stage**				<0.001				<0.001
N0		Reference				Reference		
N1a	0.805	0.724	0.894	<0.001	1.150	1.026	1.289	0.017
N1b	0.934	0.853	1.023	0.140	1.319	1.188	1.465	<0.001
N1c	0.863	0.680	1.094	0.223	1.147	.900	1.463	0.267
N2a	1.024	0.934	1.123	0.608	1.487	1.336	1.656	<0.001
N2b	1.327	1.218	1.445	<0.001	1.905	1.715	2.116	<0.001
**Colectomy**				<0.001				<0.001
Standard colectomy		Reference				Reference		
Simplified colectomy	1.245	1.142	1.358	<0.001	1.343	1.229	1.469	<0.001
Non-colectomy/NOS	1.964	1.817	2.123	<0.001	2.599	2.288	2.953	<0.001
**Hepatic surgery**				<0.001				<0.001
Yes		Reference				Reference		
No/unknown	1.971	1.807	2.150	<0.001	1.502	1.373	1.643	<0.001
**Radiotherapy**				<0.001				.100
Yes		Reference				Reference		
No/Unknown	1.476	1.319	1.651	<0.001	1.110	0.980	1.256	0.100
**Chemotherapy**				<0.001				<0.001
Yes		Reference				Reference		
No/Unknown	2.850	2.669	3.044	<0.001	2.387	2.223	2.563	<0.001
**CEA**				<0.001				<0.001
Negative		Reference				Reference		
Positive	1.697	1.532	1.880	<0.001	1.624	1.465	1.801	<0.001
NOS	1.666	1.492	1.860	<0.001	1.476	1.321	1.649	<0.001
**PNI**				<0.001				0.412
Negative		Reference				Reference		
Positive	1.091	1.011	1.178	0.025	1.043	0.964	1.129	0.293
NOS	1.403	1.297	1.518	<0.001	0.970	0.885	1.064	0.521

MCC: mucinous cell carcinoma; SRCC: signet ring cell carcinoma; RNE: regional nodes examined; PNI: perineural invasion; NOS: not otherwise specified; NA: Unavailable. †: Rectum includes Rectosigmoid junction.

**Table 3:** Univariable and multivariable Cox regression model analyses of CSS for nomogram

Characteristics	Univariable analysis				Multivariable analysis			
	OR	95% CI lower	95% CI upper	*p*-value	OR	95% CI lower	95% CI upper	*p*-value
**Gender**				0.060				
Female		Reference				NA		
Male	0.935	0.872	1.003	0.060				
**Age (years)**				<0.001				<0.001
≤50		Reference				Reference		
51-60	1.111	0.997	1.238	0.057	1.065	0.954	1.189	0.259
61-70	1.242	1.117	1.382	<0.001	1.151	1.032	1.283	0.011
71-80	1.777	1.585	1.992	<0.001	1.503	1.333	1.695	<0.001
>80	3.221	2.835	3.660	<0.001	2.070	1.790	2.395	<0.001
**Marital status**				<0.001				<0.001
Married		Reference				Reference		
Single	1.261	1.150	1.383	<0.001	1.262	1.147	1.388	<0.001
Divorced/Separated	1.142	1.018	1.281	0.024	1.083	0.964	1.217	0.181
Widowed	1.949	1.749	2.171	<0.001	1.159	1.030	1.304	0.015
NOS	1.127	0.959	1.326	0.147	1.030	.875	1.213	0.722
**Insurance**				0.857				
Yes		Reference				NA		
No/unknown	0.987	0.852	1.142	0.857				
**Race**				<0.001				<0.001
White		Reference				Reference		
Black	1.257	1.146	1.378	<0.001	1.166	1.059	1.283	0.002
Other/NOS	0.902	0.795	1.023	0.109	0.852	0.750	0.968	0.014
**Tumor location**				<0.001				<0.001
Right colon		Reference				Reference		
Left colon	0.621	0.572	0.673	<0.001	0.719	0.660	0.782	<0.001
Rectum^ †^	0.674	0.616	0.737	<0.001	0.751	0.677	0.833	<0.001
NOS	1.408	1.096	1.810	0.007	1.244	0.965	1.604	0.092
**Pathological grade**				<0.001				<0.001
I		Reference				Reference		
II	0.937	0.780	1.124	0.482	1.101	0.915	1.324	0.310
III	1.434	1.182	1.740	<0.001	1.527	1.253	1.861	<0.001
IV	1.899	1.493	2.415	<0.001	1.942	1.517	2.485	<0.001
Unknown	1.274	1.028	1.579	.027	1.089	0.873	1.359	0.451
**Histological type**				<0.001				0.017
Adenocarcinomas		Reference				Reference		
MCC/SRCC	1.315	1.142	1.514	<0.001	1.193	1.033	1.378	0.017
**T stage**				<0.001				<0.001
T1		Reference				Reference		
T2	0.412	0.325	0.522	<0.001	0.702	0.544	0.905	0.006
T3	0.548	0.489	0.613	<0.001	0.799	0.685	0.931	0.004
T4a	0.810	0.713	0.921	0.001	1.100	0.927	1.304	0.275
T4b	0.786	0.680	0.909	0.001	1.032	0.874	1.218	0.711
**N stage**				<0.001				<0.001
N0		Reference				Reference		
N1a	0.822	0.728	0.927	0.001	1.183	1.038	1.348	0.012
N1b	0.943	0.850	1.047	0.270	1.309	1.160	1.477	<0.001
N1c	0.871	0.665	1.140	0.315	1.083	0.821	1.428	0.572
N2a	1.034	0.930	1.149	0.538	1.485	1.314	1.679	<0.001
N2b	1.391	1.263	1.532	<0.001	1.989	1.765	2.241	<0.001
**Colectomy**				<0.001				<0.001
Standard colectomy		Reference				Reference		
Simplified colectomy	1.222	1.106	1.350	<0.001	1.338	1.207	1.484	<0.001
Non-colectomy/NOS	1.984	1.814	2.170	<0.001	2.714	2.349	3.136	<0.001
**Hepatic surgery**				<0.001				<0.001
Yes		Reference				Reference		
No/unknown	1.960	1.776	2.162	<0.001	1.479	1.336	1.637	<0.001
**Radiotherapy**				<0.001				0.235
Yes		Reference				Reference		
No/Unknown	0.666	0.586	0.757	<0.001	1.090	0.945	1.258	0.235
**Chemotherapy**				<0.001				<0.001
Yes		Reference				Reference		
No/Unknown	2.843	2.635	3.068	<0.001	2.412	2.221	2.620	<0.001
**CEA**				<0.001				<0.001
Negative		Reference				Reference		
Positive	1.722	1.534	1.934	<0.001	1.593	1.417	1.791	<0.001
NOS	1.702	1.502	1.929	<0.001	1.466	1.292	1.663	<0.001
**PNI**				<0.001				0.099
Negative		Reference				Reference		
Positive	1.102	1.011	1.202	0.027	1.082	0.990	1.184	0.084
NOS	1.367	1.248	1.496	<0.001	0.948	0.854	1.054	0.325

MCC: mucinous cell carcinoma; SRCC: signet ring cell carcinoma; RNE: regional nodes examined; PNI: perineural invasion; NOS: not otherwise specified; NA: Unavailable. †: Rectum includes Rectosigmoid junction.

**Table 4:** The C-indices for predictions of overall survival and cancer-specific survival

	OS		CSS	
	C-index	95% CI	C-index	95% CI
Training group	0.744	0.736-0.752	0.741	0.732-0.750
Validation group	0.749	0.738-0.760	0.753	0.741-0.766

Abbreviations: OS, overall survival; CSS, cancer-specific survival; C-index, index of concordance; CI, confidence interval.
