# Supplementary material for: Nomograms predicting Overall Survival and Cancer-specific Survival for Synchronous Colorectal Liver-limited Metastasis
Source: J Cancer. 2020 Aug 27;11(21):6213–25. doi: 10.7150/jca.46155 (PMC7532510; doi:10.7150/jca.46155)
Supplement: Supplementary file 1 — Supplementary figure S1. [file jcav11p6213s1.pdf]

Supplementary Fig.1 the survival curves compared the difference among non-colectomy, standard and simplified colectomy in patients without hepatic surgery definitely. A: The median OS were 24-month, 19-month and 14-month for standard colectomy, simplified colectomy and non-colectomy respectively. B: The median CSS were 26-month, 21-month and 16-month for standard colectomy, simplified colectomy and non-colectomy respectively.

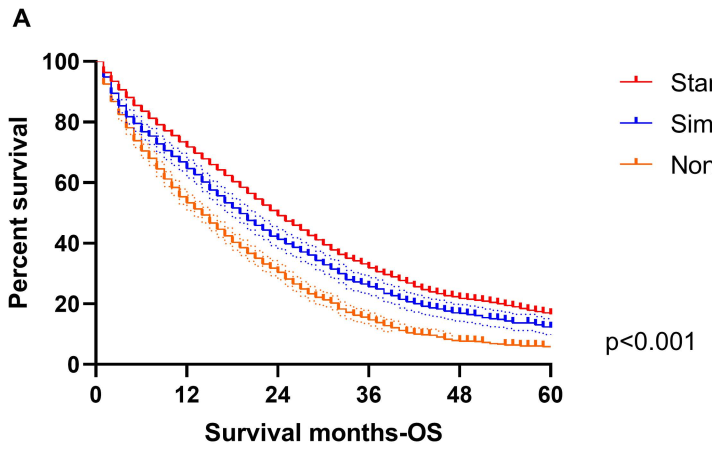
A
Percent survival
100
80
60
40
20
0
0
12
24
36
48
60
Survival months-OS
Standard colectomy
Simplified colectomy
Noncolectomy/NOS
p<0.001

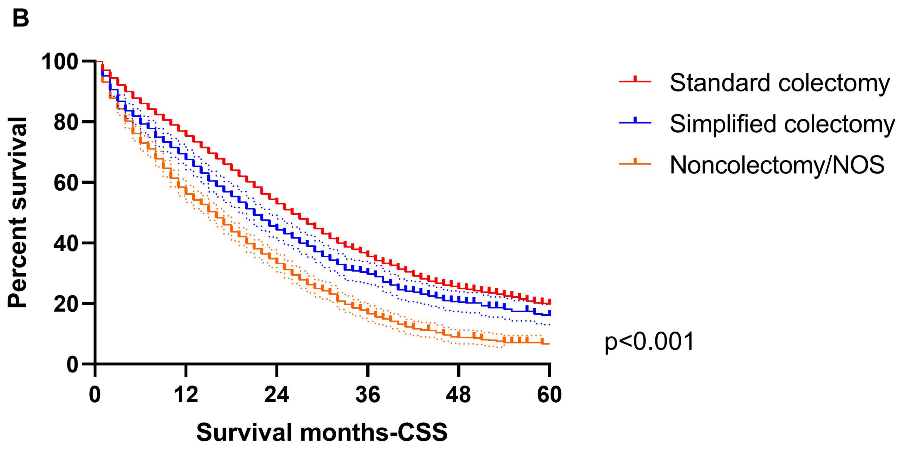
B
Percent survival
100
80
60
40
20
0
0
12
24
36
48
60
Survival months-CSS
Standard colectomy
Simplified colectomy
Noncolectomy/NOS
p<0.001
